# Investigations of Thermal, Mechanical, and Gas Barrier Properties of PA11-SiO_2_ Nanocomposites for Flexible Riser Application

**DOI:** 10.3390/polym14204260

**Published:** 2022-10-11

**Authors:** Jihong Wen, Dong Huang, Yan Li, Xichong Yu, Xinpeng Zhang, Xiaoyu Meng, Chuanbo Cong, Qiong Zhou

**Affiliations:** 1 New Energy and Material College, China University of Petroleum, Beijing 102249, China; 2 Key Laboratory of Deepwater Engineering, CNOOC Research Institute Co., Ltd., Beijing 100027, China

**Keywords:** PA11, SiO_2_, permeability, flexible riser

## Abstract

Acidic gas penetration through the internal pressure sheath of a flexible riser tends to cause a corrosive environment in the annulus, reducing the service life of the flexible riser. Nanoparticles can act as gas barriers in the polymer matrix to slow down the gas permeation. Herein, we prepared PA11/SiO_2_ composites by the melt blending method. The effect of adding different amounts of SiO_2_ to PA11 on its gas barrier properties was investigated by conducting CO_2_ permeation tests between 20 °C and 90 °C. As the temperature increased, the lowest value of the permeability coefficient that could be achieved for the PA11 with different contents of SiO_2_ increased. The composites PA/0.5% SiO_2_ and PA/1.5% SiO_2_ had the lowest permeation coefficients in the glassy state (20 °C) and rubbery state (≥50 °C). We believe that this easy-to-produce industrial PA/SiO_2_ composite can be used to develop high-performance flexible riser barrier layers. It is crucial for understanding riser permeation behavior and enhancing barrier qualities.

## 1. Introduction

**Figure 1:**
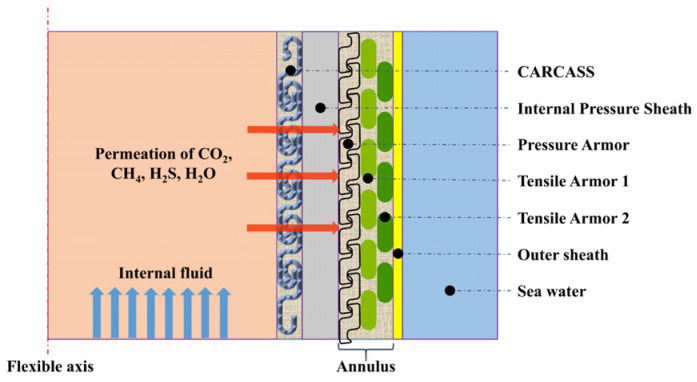
Permeation in the flexible riser.

Flexible risers are connecting pipelines that transport oil and gas from the seabed to the ground, which plays a key role in oil and gas development [1]. During the operation of the flexible risers, the internal fluid of the risers contains lots of acidic gases (CO_2_, H_2_S), as well as gas-phase water, which penetrates from the inner pressure sheath into the area between the inner and outer sheaths, a space known as the permeation annulus (Figure 1). Meanwhile, condensate or seawater can penetrate the annular space due to damage of the outer sheaths during installation. The highly corrosive environment caused by water and acidic gases poses a serious challenge to the selection of long-life internal pressure sheaths materials [2,3,4].

Commonly used polymers for internal pressure sheaths include polyamide-11 (PA11), polyvinylidene fluoride (PVDF), high-density polyethylene (HDPE), and cross-linked polyethylene (XLPE), with PA11 being the most used of the numerous materials [5]. Polyamides have good all-around properties and are one of the high-performance engineering plastics [6]. PA11 possesses outstanding mechanical characteristics (such as high fatigue resistance, a low frictional coefficient, and excellent creep resistance) and is widely used as a candidate material for flexible riser liners [7]. It also has high impact strength and remarkable chemical resistance [8], and has been used in multiple applications, including offshore pipelines, cables, automobiles, electroactive polymer actuators, and sensors [7,8,9,10]. However, PA11 needs to be modified to further improve the barrier property and to alleviate the disadvantage of poor heat resistance.

Polymer-based nanocomposites have been extensively explored for the numerous innovative and remarkable features that nanoparticles can provide to polymers [11,12,13,14]. Previous studies have shown that the performance of PA11 can be improved by nanoparticle modification [7,15]. In the flexible riser field, the elastic modulus and the hardness parameters of the PA11 matrix were enhanced by the addition of montmorillonite (MMT) [16]. The concentration of 1.0 wt.% of commercial graphene nanoplatelets (GNP) promoted improvements in the thermal and mechanical properties of PA11 [17]. Among many inorganic nanoparticles, SiO_2_ has been widely studied, due to its low price, easy availability, and high surface area. The PA11/SiO_2_ nanocomposite coatings with nominal 15 vol% hydrophobic silica attained an enhancement in scratch resistance by 35% and wear resistance by 67% compared to neat polymer coatings [15]. Although some researchers have studied the effect of different nanoparticles on polymer permeability [18,19,20,21], a single nanoparticle ratio and a single test temperature cannot accurately reflect the mechanism of nanoparticle influence on the barrier behavior of polymer-based nanocomposites.

In this paper, PA11/SiO_2_ (sphere, 25 nm, 100 m^2^/g) composites synthesized with different ratios were prepared by melt blending to evaluate their potential application in oil and gas pipelines. The CO_2_ permeability of the PA11/SiO_2_ composites at different temperatures was measured to explore the mechanism of the effect of different nanoparticle ratios on the permeation behavior of the polymer matrix. The results of the study are important for understanding riser permeation behavior and improving barrier properties, and can provide a reference for the prediction of the flexible riser annulus under various working circumstances.

## 2. Materials and Methods

### 2.1. Materials

Polyamide 11 resin (PA11, Rilsan^®^ BESNO P4) was supplied by Arkema (Suzhou, China). Hydrophobic gas-phase SiO_2_ powder (sphere) with a particle size of 25 nm and a specific surface area of 100 m^2^/g was supplied by MACKLIN (Beijing, China).

### 2.2. Fabrication of PA11/SiO_2_ Composites

First, the PA11 and SiO_2_ were vacuum-dried at 70 °C for 24 h, after which they were melt blended at 205 °C and 50 rpm for 10 min at a certain weight ratio using a mixer (Extruder, Brabender GMBH&Co.KG, Duisburg, Germany). The samples were then pre-pressed at 205 °C and 200 bar pressure for 5 min using a flatbed vulcanizing machine (LHHS 20-170919A, Lina Industrial Co., Ltd., Guangdong, China). Eventually, the PA11/x% SiO_2_ (x = 0.5, 1.0, 1.5, 2.0.) composite samples were obtained by continued cold pressing at 25 °C and 100 bar for 10 min, where x% represents the mass fraction of the SiO_2_ in PA11.

### 2.3. Morphology Characterization

Scanning electron microscopy (SEM, SU8010, HITACHI, Hitachi City, Japan) and Transmission Electron Microscopy (TEM, Tecnai G2 F20, FEI, Sarum, OR, USA) were conducted to analyze the morphology of the prepared composites. Ultramicrotome (EM UC6, Leica, Wetzlar, Germany) was used to prepare the specimens with a thickness of ~100 nm for the TEM investigation.

### 2.4. Structure Characterizations

Infrared spectra of the composites were recorded with a Fourier transform infrared spectrometer (FTIR, Tensor II, Bruker, Billica, MA, USA) between 4000 and 600 cm^−1^. X-ray diffraction (XRD) analysis was conducted on a Bruker D8 Focus X-ray diffractometer by a solid detector and Cu Ka radiation. The 2θ ranges were from 5° to 40° at a rate of 5°/min.

### 2.5. Thermal Analysis

The non-isothermal crystallization behavior in the melts of the composites were performed by using a differential scanning calorimeter (DSC, 204F1, Netzsch, Berlin, Germany). To eliminate thermal history, the samples were first heated from 30 to 205 °C at 20 °C min^−1^, held at the high temperature for 5 min, and then cooled to 20 °C at 10 °C min^−1^. After this thermal treatment, the samples were heated from 20 °C to 200 °C at 10 °C min^−1^ for analysis (2nd scan). The crystallization temperature (*T*_c_) and the enthalpy of crystallization (Δ*H*_c_) were measured during the cooling scan. The melting temperature (*T*_m_) and the enthalpy of fusion (Δ*H*_m_) were measured during the 2nd scanning process. The crystallinity (*X*_m_) was estimated according to Equation (1):

(1) $$X_{m}(\%)=(\frac{\Delta H_{m}}{\Delta H_{0}})\times 100\%$$

where Δ*H*_m_ and Δ*H*_0_ denote the heats (J/g) of melting of the PA11/SiO_2_ composites and PA11 crystals of infinite size with a value of 225.9 J/g [22].

Thermogravimetric analysis (TGA) was conducted with a DTA-TG apparatus (DTG-60, SHIMADZU, Kyoto, Japan) ranging from 50 °C to 700 °C with a heating rate of 10 °C/min in a N_2_ atmosphere (flow rate 100 mL min^−1^).

### 2.6. Mechanical Properties

The mechanical performance was obtained with an Instron machine (WDL-5000N, Daochun, Yangzhou, China) at a strain speed of 50 mm min^−1^. The samples were cut into 15 × 2 mm dumbbell shapes.

Dynamic mechanical analysis (DMA) was performed using a DMA apparatus (Q800, TA INSTRUMENTS, New Castle, PA, USA) over a temperature range of −20 °C to 100 °C (3 °C min^−1^, 1 Hz), which covers the temperature range of the PA11 used for flexible riser inner sheaths. The tests were carried out in tension mode. The DMA measurement was used to determine the viscoelastic properties of the tested materials, such as storage modulus (E′), loss modulus (E″), and damping parameter (tan δ) [23].

### 2.7. Gas Barrier Test

The applicable temperature range of PA11 is from −20 °C to90 °C, according to ISO 13628-2017 (Petroleum and natural gas industries—design and operation of subsea production systems Part 11: Flexible pipe systems for subsea and marine applications). The samples with a thickness of approximately 0.5 mm were placed in the permeability unit test apparatus. The low-pressure ends of the composite materials were supported by breathable steel, so that the increase in pressure would not cause deformation failure. The experimental temperature was far lower than the aging temperature of the materials, which will not cause material failure. The input pressure for the test was 4 MPa, and the test temperatures were 20 °C, 30 °C, 50 °C, 70 °C, and 90 °C. Gas permeation tests were conducted according to the corresponding standard GB/T 40260-2021 (Test method for determining gas permeability of polymer membrane materials), and different batches of the samples were used to test the gas permeability.

**Figure 2:**
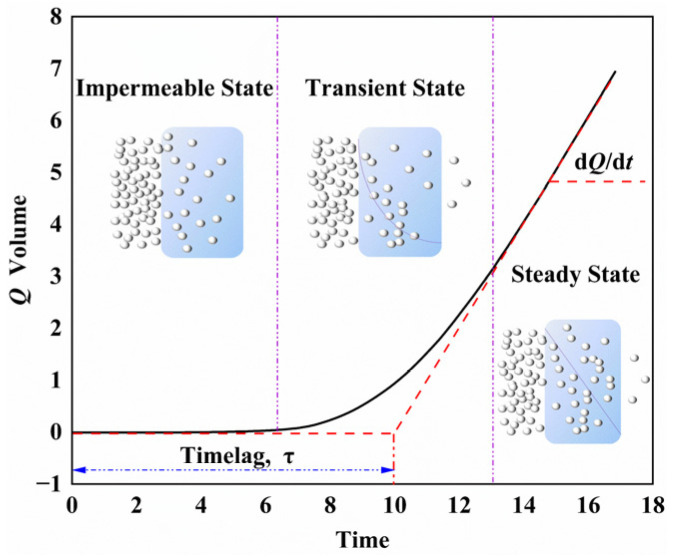
Gas permeate volume curve with time.

The permeation of gas molecules into the polymer was divided into three stages [24], as shown in Figure 2. The first stage was the impermeable state, where the gas molecules collided with the polymer surface. After the transient (unsteady) permeation state, the steady-state permeation state was finally reached, where the diffusion was stable, due to the complete saturation of the internal volume. The permeability coefficient (*P*) and diffusion coefficient (*D*) were calculated according to the permeability curve through Equations (2) and (3), respectively [25,26,27]:

(2) $$\frac{d_{Q}}{d_{t}}=P\times A\times (P_{1}-P_{2})/h$$

(3) $$D=\frac{h^{2}}{6\tau }$$

where *A* denotes the permeated area, *P*_1_ and *P*_2_ stand for high-end and low-end pressure, and *h* and *τ* refer to the thickness of the samples and lag time, respectively.

## 3. Results and Discussion

### 3.1. Morphology Characterization

**Figure 3:**
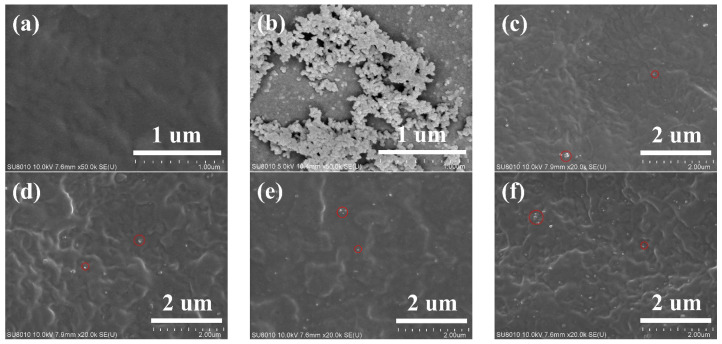
SEM images of composite with different SiO_2_ contents: (**a**) PA11, (**b**) SiO_2_, (**c**) PA11/0.5% SiO_2_, (**d**) PA11/1.0% SiO_2_, (**e**) PA11/1.5% SiO_2_, (**f**) PA11/2.0% SiO_2_.

SEM images for the pure PA11 and the PA11/SiO_2_ composite cross-sections are shown in Figure 3. The distribution of the SiO_2_ in the PA11 matrix was almost uniform with less agglomeration (the red circle in Figure 3c–f). In addition, the SEM image shows that the sample was flat and free of defects, which ensured the reliability of the mechanical properties and gas permeate behavior tests.

**Figure 4:**
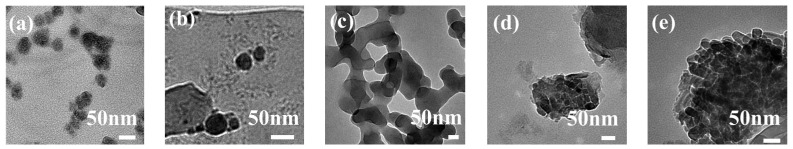
TEM images PA11/SiO_2_ composite, (**a**) SiO_2_, (**b**) PA11/0.5% SiO_2_, (**c**) PA11/1.0% SiO_2_, (**d**) PA11/1.5% SiO_2_, (**e**) PA11/2.0% SiO_2_.

TEM was performed to further investigate the distribution morphology of the SiO_2_. The SiO_2_ nanoparticles had a round structure and a size of 25–35 nm, as shown in Figure 4a. For the PA11/SiO_2_ composites, the dispersion of the SiO_2_ particles in the PA11 matrix was more uniform when the addition amount of nanoparticles was low (below 1.0%), as shown in Figure 4b,c. When the addition was increased to 1.5%, there was a certain degree of nanoparticle agglomeration in the PA11, with a size of about 5 μm, as shown in Figure 4d.

### 3.2. DSC Characterization

**Figure 5:**
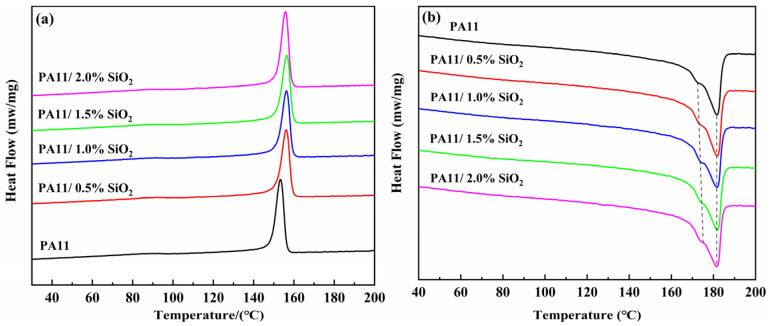
DSC curves of PA11/SiO_2_ composites: (**a**) crystallization curve, (**b**) melting curve.

It was assumed that the gas penetration in the polymers occurred in the amorphous zone rather than in the crystalline region, which is a limited region [28]. To investigate the impact of the SiO_2_ incorporation on the melting and crystallization behaviors of the PA11, DSC tests were performed on the pure PA11 and PA11/SiO_2_ composites, as shown in Figure 5. As summarized in Table 1, the *T*_c_ of the neat PA11 was 153.3 °C. With the addition of the SiO_2_ nanoparticles, the *T*_c_ values of the composites improved, demonstrating that SiO_2_ acted as a nucleating agent for the PA11 crystallization and increased the crystallization rate [29,30]. Furthermore, the addition of a small amount of SiO_2_ had no significant effect on the crystallinity of the PA11.

This was mainly attributed to the potent intrinsic hydrogen bonding network that is typical of polyimides, which stabilized the PA11 amorphous phase [31]. For melting curves, all PA11/SiO_2_ composite samples showed two melting peaks, which can be attributed to the fusion of different types of crystals in the semi-crystallized PA11. The melting peaks at higher temperatures might be attributed to the melting of the perfect crystals, whereas the melting peaks at lower temperatures could be assigned to the deficient crystals produced by short molecular chains [32,33].

### 3.3. Structure Characterization

**Figure 6:**
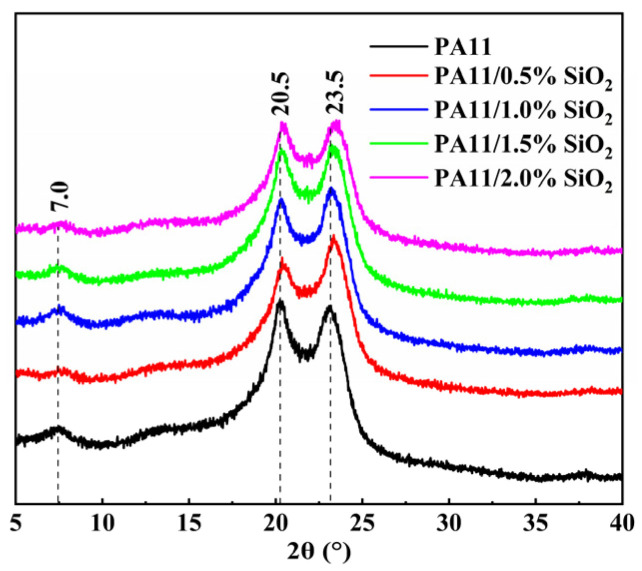
XRD spectra of PA-11 and PA11/SiO_2_ composites.

The crystal structures of the PA11 and PA11/SiO_2_ nanocomposites were characterized by XRD, as presented in Figure 6. The main crystalline peaks in the XRD spectra were observed at 2θ = 7°, 20.5°, and 23.5°, which corresponded to the α-polymorph of the PA11 [34]. Moreover, the addition of the SiO_2_ did not affect the crystal structure of the PA11.

**Figure 7:**
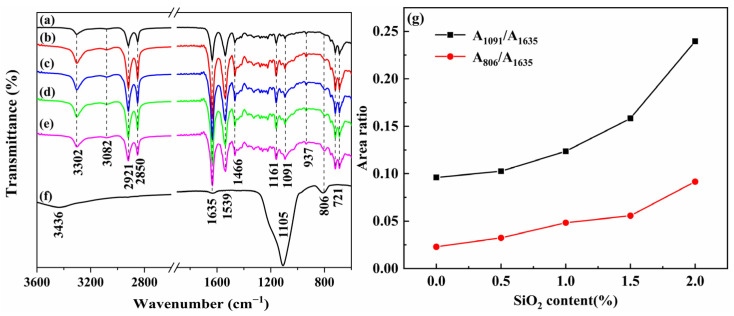
FTIR spectra of PA11 and PA11/SiO_2_ composites: (**a**) PA11, (**b**) PA11/0.5% SiO_2_, (**c**) PA11/1.0% SiO_2_, (**d**) PA11/1.5% SiO_2_, (**e**) PA11/2.0% SiO_2_, (**f**) SiO_2_, (**g**) A_1091_/A_1635_ and A_806_/A_1635_ ratios of FTIR peak areas.

The structures of the PA11, SiO_2_, and PA11/SiO_2_ composites were characterized by FTIR (Figure 7). In the curve of the PA11, the bands at 1635 and 1539 cm^−1^ corresponded to the C=O stretching vibration and N-H bending vibration, respectively [6,35,36,37,38,39,40]. The peaks at 806 cm^−1^ and 1105 cm^−1^ indicated the anti-symmetric stretching and asymmetric stretching vibrations of the Si-O-Si, respectively [41,42]. The area ratio of absorption bands at 1091 cm^−1^ (A_1091_) and 806 cm^−1^ (A_806_) to 1635 cm^−1^ (A_1635_) exhibited the relative amounts of Si-O-Si groups in the composites. As shown in Figure 7g, compared with the PA11, the peak area ratios of the A_1091_/_1635_, and A_806_/_1635_ increased with the SiO_2_ additive amount, which demonstrated the successful introduction of the SiO_2_ into the PA11 matrix.

### 3.4. Mechanical and Thermal Stability Characterization

**Figure 8:**
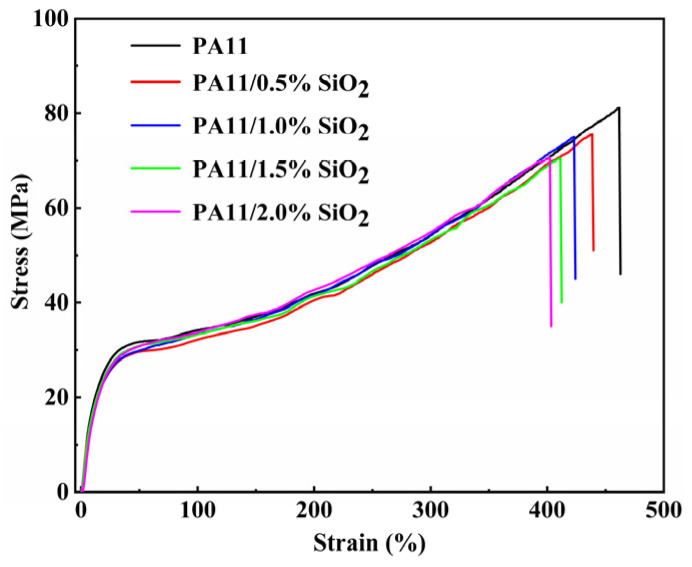
The stress–strain curves of PA11 and PA11/SiO_2_ composites.

Figure 8 depicts the stress–strain curves of the PA11 and PA11/SiO_2_ composites. The mechanical properties determined from the curves are shown in Table 2. The neat PA11 had a tensile strength of 78.4 MPa, an elasticity modulus of 89.9 MPa, and a break elongation of 447.7%. As the SiO_2_ content increased, the nanoscale SiO_2_ aggregated and existed in the form of defects in the PA11/SiO_2_ composites, which reduced the tensile strengths and break elongations of the composites to some extent. When compared to the pristine PA11, the presence of SiO_2_ in the PA11 resulted in a higher elasticity modulus and a decrease in the break elongation. The findings are consistent with previous research [43,44].

**Figure 9:**
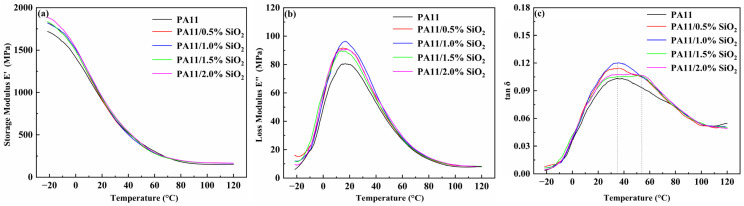
(**a**) Storage modulus, (**b**) loss modulus, and (**c**) tan δ of neat PA11 along with PA11/SiO_2_ composites.

The mechanical properties of the PA11 and nanocomposites were further studied by dynamic mechanical analysis (DMA). The changes in the energy storage modulus (E′) and loss modulus (E″) with temperature are shown in Figure 9. E′ and E″ increased above or below the glass transition temperature (*T*_g_). Changes below the *T*_g_ were greater than those above the *T*_g_. This result was consistent with other studies on polymer nanocomposites reinforced by nanomaterials [30,45].

When the PA11 was in the glassy state rather than the rubber state, the effect of the nanoparticles was more significant. The relaxation of the PA11 and nanocomposites was studied using tan δ data shown in Figure 9c. For the *T*_g_-associated *α* relaxation centered at 35–40 °C, there was a transition to a higher temperature in many nanocomposite samples. The SiO_2_ may have reduced the free volume in the PA11 by hindering the chain movement and conformation change. In addition, at the high SiO_2_ content in Figure 9c, the tan δ peak became wider, and a second higher *T*_g_ was observed, showing that the molecular chain movement of the PA11 was restricted, resulting in a large realistic response of the nanocomposites [46,47,48].

**Figure 10:**
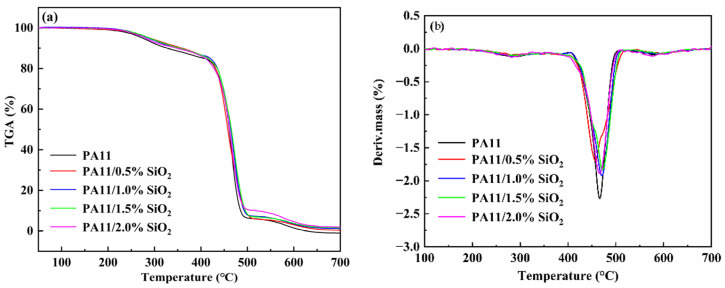
TGA curves (**a**) and DTG curves (**b**) of neat PA11 and PA11/SiO_2_ composites.

TGA and DTG were performed to test the thermal stability of the neat PA11 and the nanocomposites with nitrogen airflow (Figure 10a,b). All the tested materials showed two main weight losses, at 280 °C and 460 °C. The first degradation step, with a shallow peak in the DTG curve centered at around 280 °C, was attributed to the decarboxylation process [49]. In the second degradation step, a main peak was identified in the DTG curves. On the other hand, the melting temperature of SiO_2_ was over 1600 °C. Thus, it was inferred that the degradation occurred due to the decomposition of the PA11 backbones.

### 3.5. CO_2_ Permeability Characterization and Behavior Analysis

**Figure 11:**
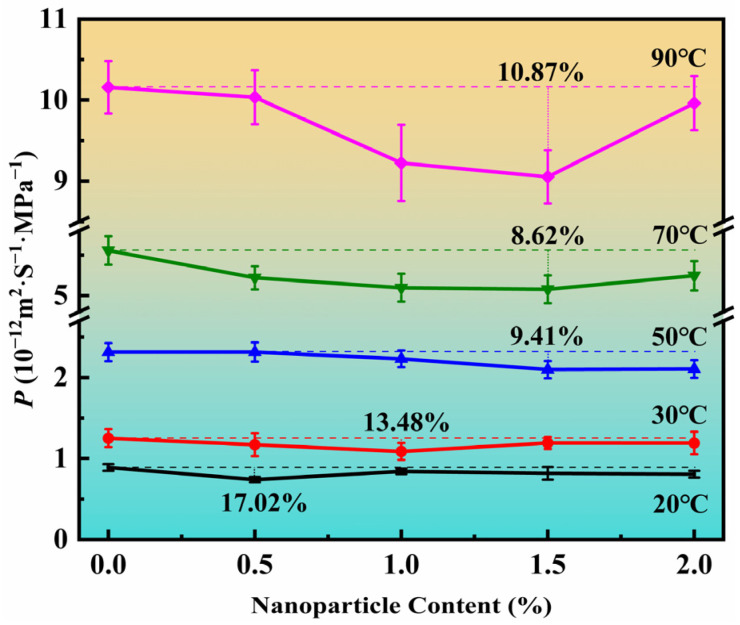
Permeability coefficient of PA11 and PA11/SiO_2_ composites at 20 °C, 30 °C, 50 °C, 70 °C, and 90 °C.

For vertical two-phase flow in pipes, the common flow patterns are annular flow, slug flow, bubble flow, and dispersed bubble flow [50]. The multiphase flow pattern has a significant effect on the temperature and pressure distribution of the flexible riser, which in turn affects the rate of gas phase permeation into the annulus within the flexible riser. Flexible risers are usually required for long-term service and therefore require an in-depth study of the steady-state permeation processes. The CO_2_ permeabilities of the PA11 and PA11/SiO_2_ composites were measured at 20 °C, 30 °C, 50 °C, 70 °C, and 90 °C. These temperatures were chosen because they are typical values for PA11 applications in flexible risers. In addition, the design of the temperatures involved two states of the PA11, the glassy state and the rubbery state. Figure 11 displays the results of the permeation coefficients for the PA11/SiO_2_ composites, each averaged over four parallel specimens from different batches. The test data error of different samples in the same batch and the error of different samples was calculated, which can meet the requirement of 5.0% of the standard.

The percentage reduction in the permeability coefficient of the PA11/SiO_2_ composites compared to the pure PA11 varied at different temperatures. The maximum decreases in the permeability coefficients of the PA11/SiO_2_ composites at 20–90 °C were: 17.02% at 20 °C, 13.48% at 30 °C, 9.41% at 50 °C, 8.62% at 70 °C, and 10.87% at 90 °C. It is evident that the percentage difference between the PA11 and PA11/SiO_2_ permeability coefficients decreased with increasing temperature. Additionally, as the temperature increased, the increase of nanoparticle content had a greater impact on the permeation behavior of the composites.

To further analyze the influence of the various nanoparticle contents on the behavior of the gas permeation at various temperatures, the activation energies for the diffusion, solubility, and permeation of the composites of PA11/SiO_2_ were calculated based on the widely used solution-diffusion model [51,52]. The effect of the temperature on gas diffusion and solubility was expressed by the Arrhenius Equations (4) and (5), respectively [28]:

(4) $$D=D_{0}\times \exp\left(-\frac{E_{D}}{RT}\right)$$

(5) $$S=S_{0}\times \exp\left(-\frac{\Delta H_{S}}{RT}\right)$$

where *D*_0_ is the pre-exponential factor and *E*_D_ is the activation energy of diffusion; Δ*H*_S_ is the partial molar enthalpy of sorption. *T* is the temperature (K), and *R* is the ideal gas constant. Hence, the permeability can be obtained by multiplying the solubility coefficient (a thermodynamic coefficient) by the diffusivity coefficient (a kinetic coefficient), and the gas permeability as a function of temperature was obtained using Equation (6) [53,54,55]:

(6) $$P=P_{0}\times \exp\left(-\frac{E_{P}}{RT}\right)$$

where *P*_0_ is the pre-exponential factor and *E*_P_ is the activation energy of permeation. In general, permeability increases with increasing temperature. The values of *E*_D_ and *E*_P_ were determined for each gas from the slopes of the ln(*D*) and ln(*P*) vs. 1000/T curves (Figures S1 and S2. Tables S1 and S2), respectively. The distinction between the *E*_P_ and *E*_D_ was represented by Δ*H*_S_ and is summarized in Table 3.

*E*_D_ increased with the increase in the nanoparticle content, indicating the diffusion of gas molecules was more difficult. Δ*H*_S_ was negative, showing that gas dissolution was an exothermic process, whereas *E*_P_ and *E*_D_ were positive, suggesting that the permeation and diffusion were heat-absorbing processes. As temperature increased, the permeability and diffusion coefficients of the PA11 to CO_2_ increased, whereas the solubility coefficient exhibited the opposite trend, as shown in Figure S3.

Gas permeation is closely related to the state of motion of the polymer. The free volume theory of gas diffusion in glass polymers proposed by Vrentas and Duda [57,58] assumes the existence of pores, i.e., free volumes, between polymer chain segments. Gas molecules jump from one free volume unit to the next to complete the diffusion process. To achieve this process, gas molecules need to have sufficiently large free volume units in adjacent positions and sufficient energy to overcome the jump resistance. Figure S4 depicts the variation pattern of the free volume with temperature and shows an obvious distinction in the permeation curves of the composites in the glass and rubber states. When the polymer was in the glassy state, the chain segment motion was in a frozen state, and no conformational changes could occur; the chain segment spacing formed during the thermal movement of the molecular chain segments was the main channel for gas diffusion [59].

**Figure 12:**
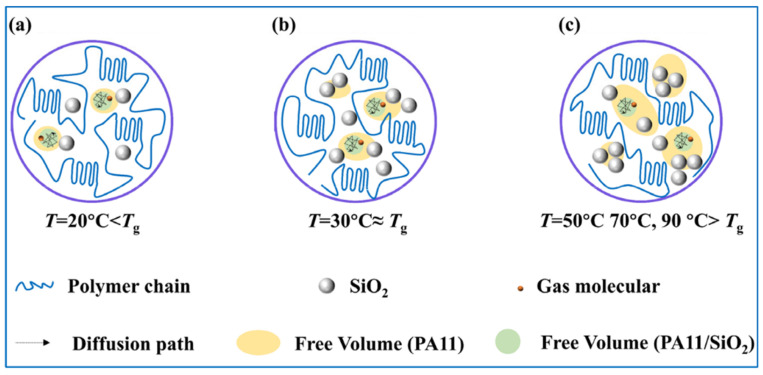
Diffusion paths of gas molecules and influence of nanoparticles on free volume at different temperatures, (**a**) PA11/0.5% SiO_2_, (**b**) PA11/1.0% SiO_2_, (**c**) PA11/1.5% SiO_2_.

In the glassy state, gas molecules had high resistance to jumping between free volumes, and the solubility had a large effect on the permeability coefficient. As shown in Figure 4, the 0.5% proportion of nanoparticles efficiently dispersed and occupied the free volume of the PA11, thus reducing the free volume (Figure 12a) and solubility. The uniformly dispersed nanoparticles could also simultaneously extend the diffusion paths of the gas molecules. As a result, the highest reduction in the PA11/0.5% SiO_2_ composite permeability coefficient occurred at 20 °C below the glass transition temperature. As the nanoparticle content increased, the nanoparticles began to agglomerate and fill the free volume, resulting in gaps and increasing solubility. The Δ*H*_S_ values in Table 3 showed the same pattern.

When the temperature gradually increased, the chain segments were unfrozen from the frozen state. In the rubbery state case, the free volume increased, and the gas molecules had low resistance to jumping. The larger free volume meant that more nanoparticles were needed to effectively inhibit the increase in permeability caused by the increase in free volume (Figure 12c). The *E*_D_ increased as the nanoparticle content rose, indicating that diffusion was difficult, and that the presence of nanoparticles slowed down the movement of the molecular chains while extending the diffusion path. When the content of nanoparticles was greater than 1.5%, the activation energy decreased, due to agglomeration diffusion. Therefore, the PA11/1.5% SiO_2_ composite exhibited the lowest permeability coefficient in the rubbery state.

## 4. Conclusions

In this study, the PA11/SiO_2_ composites were made by vacuum densification and mixing in a range of proportions. The DMA results demonstrated that the presence of nanoparticles impeded the mobility of the molecular chains. It is evident from the CO_2_ permeation performance tests carried out at 20 °C, 30 °C (the glassy state), 50 °C, 70 °C, and 90 °C (the high elasticity state) that when the temperature rose from 20 °C to 90 °C, the amount of SiO_2_ that displayed the lowest permeability coefficient rose. When the composites were in a glass state, the solubility had the greatest influence on the permeability behavior. The uniformly dispersed nanoparticles made the molecular diffusion path longer, which had an obvious blocking effect. At 20 °C, the permeability coefficient of the PA11/0.5% SiO_2_ composite decreased by 17.02% compared with that of the PA11. When the composite was in the rubber state, more nanoparticles were required to inhibit the permeability increase caused by the increase in free volume. However, when the nanoparticles aggregated, the diffusion coefficient increased. Therefore, the permeability coefficient of the PA11/1.5% SiO_2_ composite in the rubbery state was the lowest. The PA11/SiO_2_ composites exhibited strong potential for use as flexible risers with high barrier performance.

## Figures and Tables

**Table 1:** DSC thermodynamic parameters of PA11/SiO_2_ composites.

Sample	*T*_c_/°C	Δ*H*_c_/J·g^−1^	*T*_m_/°C	Δ*H*_m_/J·g^−1^	*X*_c_/%
PA11	153.3	52.50	181.7	53.39	23.63
PA11/0.5% SiO_2_	156.1	54.00	181.8	54.50	24.13
PA11/1.0% SiO_2_	156.3	51.67	181.7	52.23	23.13
PA11/1.5% SiO_2_	156.4	52.98	181.9	53.67	23.76
PA11/2.0% SiO_2_	155.8	53.48	181.7	54.54	24.15

**Table 2:** Mechanical properties of PA11/SiO_2_ composites.

Sample	Tensile Strength (MPa)	Elongation at Break (%)	Elasticity Modulus (MPa)
PA11	78.5 ± 3.6	447.7 ± 12.7	89.9 ± 4.6
PA11/0.5%SiO_2_	74.1 ± 2.3	425.6 ± 16.6	92.7 ± 9.8
PA11/1.0%SiO_2_	73.9 ± 3.0	413.8 ± 9.9	97.2 ± 7.4
PA11/1.5%SiO_2_	72.0 ± 2.7	406.9 ± 12.5	97.9 ± 3.7
PA11/2.0%SiO_2_	71.0 ± 2.1	399.1 ± 13.5	97.4 ± 8.8

**Table 3:** Apparent activation energies for permeability, diffusion, and heat of solution of CO_2_ in PA11.

Samples	PA11	PA11/ 0.5% SiO_2_	PA11/ 1.0% SiO_2_	PA11/ 1.5% SiO_2_	PA11/ 2.0% SiO_2_	Reference
*E*_P_ (kJ/mol)	31.94	32.81	30.63	30.99	31.08	35 [28], 34 [56]
*E*_D_ (kJ/mol)	45.74	46.17	46.22	50.79	48.40	40 [28], 52 [56]
Δ*H*_S_ (kJ/mol)	−13.80	−13.36	−15.58	−19.80	−17.31	(−10)–(−4) [28] (−18)–(−13) [56]

## Data Availability

Not applicable.

## Supplementary Materials

The following supporting information can be downloaded at: https://www.mdpi.com/article/10.3390/polym14204260/s1, Figure S1: Temperature influence on transport coefficients of CO_2_ in PA11/SiO_2_ composite, Permeability versus reciprocal temperature; Figure S2: Temperature influence on transport coefficients of CO_2_ in PA11/SiO_2_ composite, diffusion versus reciprocal temperature; Figure S3: Permeability coefficient, diffusion coefficient, and solubility of CO_2_ in PA11 vary with temperature; Figure S4: Permeation curve of PA11 at 20 °C, 30 °C, 50 °C, 70 °C, and 90 °C, diagram of free volume with temperature; Table S1: Intercept and slope of permeability–temperature fitting curve; Table S2: Intercept and slope of diffusion–temperature fitting curve.
