# Supplementary material for: Investigations of Thermal, Mechanical, and Gas Barrier Properties of PA11-SiO2 Nanocomposites for Flexible Riser Application
Source: Polymers (Basel). 2022 Oct 11;14(20):4260. doi: 10.3390/polym14204260 (PMC9610365; doi:10.3390/polym14204260)
Supplement: Supplementary file 1 [file polymers-14-04260-s001.zip › polymers-1924040-supplementary.pdf]

Supporting File

Investigations of Thermal, Mechanical, and Gas Barrier Properties of PA11-SiO_2 Nanocomposites for Flexible Riser Application

Jihong Wen [1], Dong Huang [1], Yan Li [2], Xichong Yu [2], Xinpeng Zhang [1], Xiaoyu Meng [1], Chuanbo Cong [1], and Qiong Zhou [1,*]

[1] New Energy and Material college, China University of Petroleum-Beijing. Beijing, 102249, China
[2] Key Laboratory of Deepwater Engineering, CNOOC Research Institute Co., Ltd. No. 6 Taiyanggong South Street, Beijing, 100027, China
* Correspondence: zhouqiong@cup.edu.cn

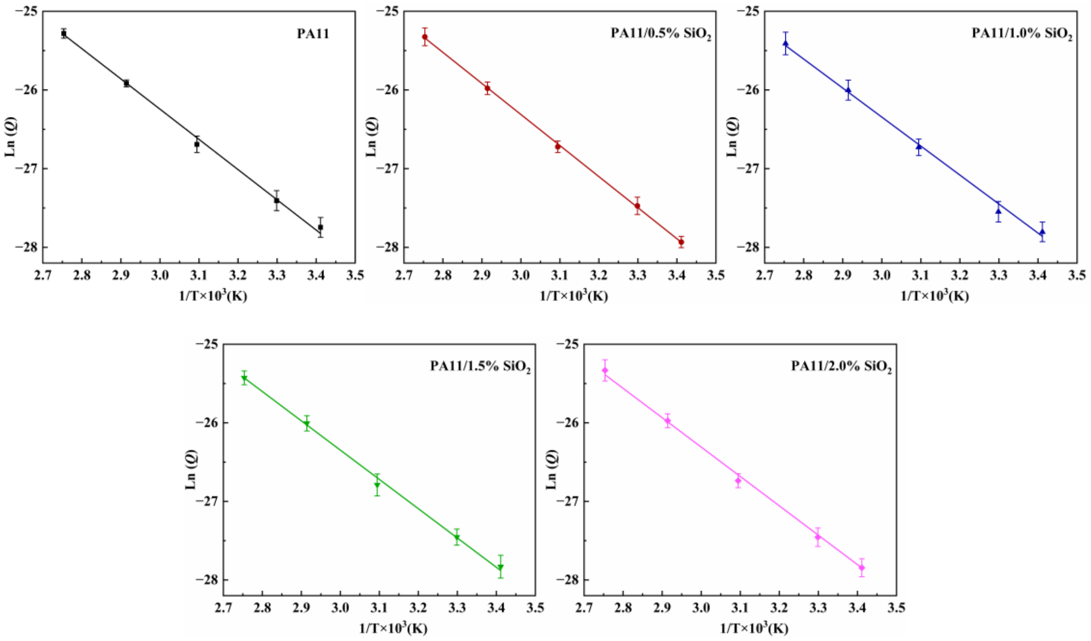

Figure S1 Temperature influence on transport coefficients of CO_2 in PA11/SiO_2 composite, Permeability versus reciprocal temperature

Table S1 Intercept and slope of permeability - temperature fitting curve

Samples	PA11	PA11/0.5%SiO_2	PA11/1.0%SiO_2	PA11/1.5%SiO_2	PA11/2.0%SiO_2
Intercept	−14.72	−14.47	−15.29	−15.16	−15.09
Slope	−3841.74	−3946.74	−3684.95	−3727.81	−3738.94

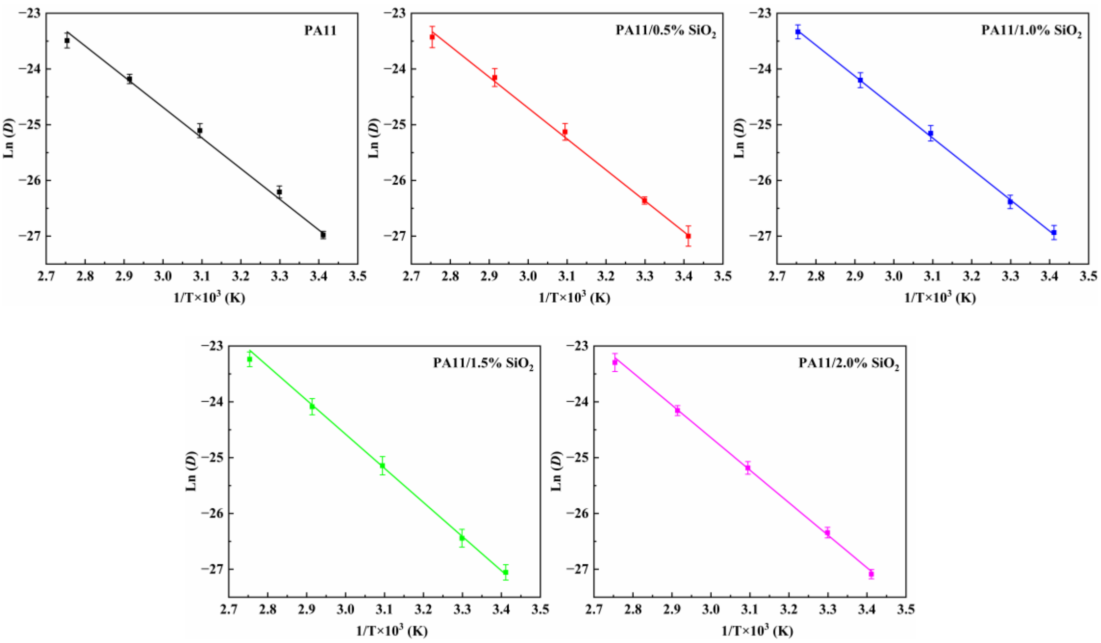

Figure S2 Temperature influence on transport coefficients of CO_2 in PA11/SiO_2 composite, Diffusion versus reciprocal temperature

Table S2 Intercept and slope of Diffusion - temperature fitting curve

**Samples**	**PA11**	**PA11/0.5%SiO_2**	**PA11/1.0%SiO_2**	**PA11/1.5%SiO_2**	**PA11/2.0%SiO_2**
Intercept	−8.18	−8.04	−8.01	−6.25	−7.18
Slope	−5501.86	−5553.82	−5559.28	−6109.72	−5821.6

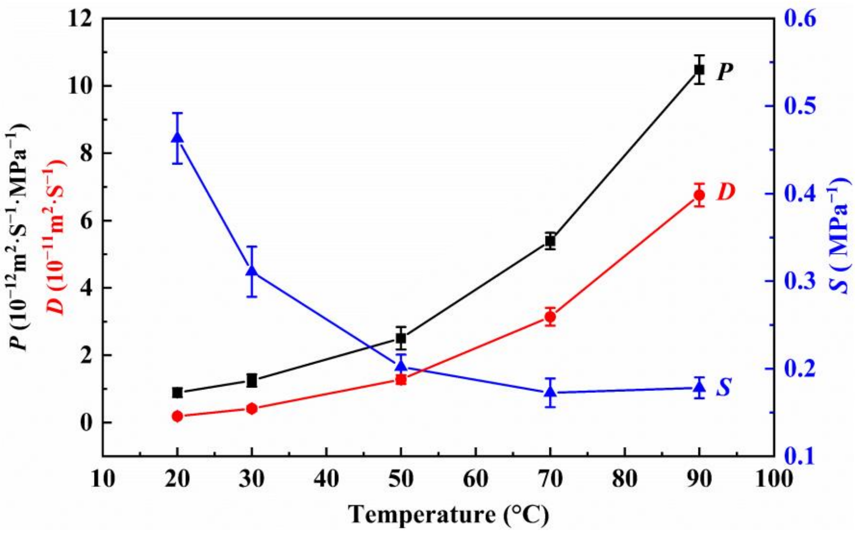

Figure S3 Permeability coefficient, diffusion coefficient, and solubility of CO_2 in PA11 vary with temperature

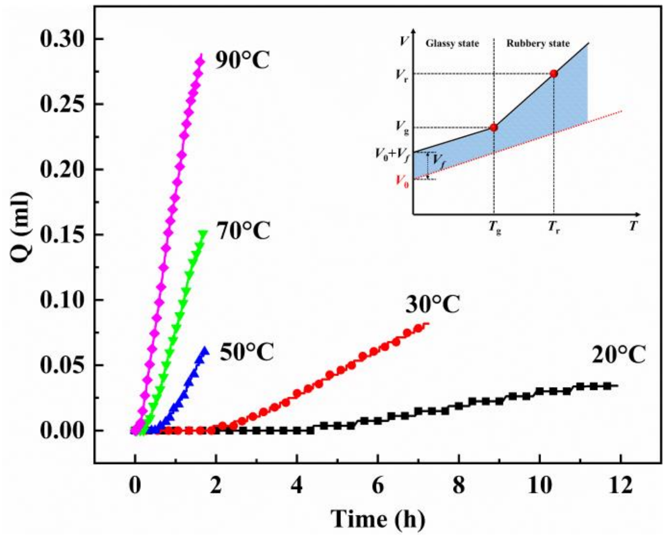

V_0-Occupied volume, V_g-The total volume in T_g temperature,

V_f-The free volume below T_g temperature, V_r-The volume at a temperature higher than T_g

Figure S4 Permeation curve of PA11 at 20°C, 30°C, 50°C, 70°C, and 90°C, diagram of free volume with temperature
